# Supplementary material for: The genome sequence of the Pine Carpet, Thera firmata Hübner, 1822 (Lepidoptera: Geometridae)
Source: Wellcome Open Res. 2025 Oct 21;10:586. [Version 1] doi: 10.12688/wellcomeopenres.25046.1 (PMC12770886; doi:10.12688/wellcomeopenres.25046.1)
Supplement: Peer review history

# Reviewer report: Reviewer response for version 1

**DOI:** 10.21956/wellcomeopenres.27612.r140058

**Published:** 2026-01-05

**Authors:** Sara Goodwin

**Affiliations:** 1 Cold Spring Harbor Laboratory, New York, USA

Review of “The genome sequence of the Pine Carpet , *Thera firmata* (Hubner, 1822) (Lepidoptera Geometridae)

 ”

 The is a data in the in The Darwin Tree of Life Project.  It is a genome assembly from a single male individual.   The report notes a high-quality, near chromosome-scale assembly for the Pine Carpet moth.   Both   PacBio HiFi Arima Hi-C were used to generate a phased assembly with two haplotype.  Haplotype 1  achieved   98.51% scaffolding across 19 pseudo molecules, while haplotype 2 is assembled to the scaffold level. . Raw data and assemblies have been deposited at ENA under PRJEB74679

 It may be useful to explain why the k-mer completeness of individual haplotypes ~81.9%  but 95.51% combined. While unsurprising, it may be confusing for some readers.

 There is a typo in the first paragraph of the methods section “Beinn Eighe National Nature Reserve National Nature Reserve”

 Review of “The genome sequence of the Pine Carpet , *Thera firmata* (Hubner, 1822) (Lepidoptera Geometridae)

 ”

 The is a data in the in The Darwin Tree of Life Project.  It is a genome assembly from a single male individual.   The report notes a high-quality, near chromosome-scale assembly for the Pine Carpet moth.   Both   PacBio HiFi Arima Hi-C were used to generate a phased assembly with two haplotype.  Haplotype 1  achieved   98.51% scaffolding across 19 pseudo molecules, while haplotype 2 is assembled to the scaffold level. . Raw data and assemblies have been deposited at ENA under PRJEB74679

 It may be useful to explain why the k-mer completeness of individual haplotypes ~81.9%  but 95.51% combined. While unsurprising, it may be confusing for some readers.

 There is a typo in the first paragraph of the methods section “Beinn Eighe National Nature Reserve National Nature Reserve”

Are sufficient details of methods and materials provided to allow replication by others?

Yes

Is the rationale for creating the dataset(s) clearly described?

Yes

Are the datasets clearly presented in a useable and accessible format?

Yes

Are the protocols appropriate and is the work technically sound?

Yes

Reviewer Expertise:

Plant and Animal genomics and sequencing technologies

I confirm that I have read this submission and believe that I have an appropriate level of expertise to confirm that it is of an acceptable scientific standard.

# Reviewer report: Reviewer response for version 1

**DOI:** 10.21956/wellcomeopenres.27612.r140060

**Published:** 2026-01-02

**Authors:** Alena Suchackova Bartonova

**Affiliations:** 1 Biology Centre of the Czech Academy of Sciences, České Budějovice, Czech Republic

This is a note for the genome sequence of the Pine Carpet moth, Thera firmata, produced by the Darwin Tree of Life initiative. The sample preparation and genome production follow standard procedures. Links to all relevant protocols and the produced data are provided. I have not spotted anything to change.

Are sufficient details of methods and materials provided to allow replication by others?

Yes

Is the rationale for creating the dataset(s) clearly described?

Yes

Are the datasets clearly presented in a useable and accessible format?

Yes

Are the protocols appropriate and is the work technically sound?

Yes

Reviewer Expertise:

entomology, butterflies, ecology, biogeography, population genetics

I confirm that I have read this submission and believe that I have an appropriate level of expertise to confirm that it is of an acceptable scientific standard.
